# Separation Practices in Children and Adolescents Admitted for Suicidal Behavior: A National Survey of French Psychiatrists

**DOI:** 10.3389/fped.2022.860267

**Published:** 2022-07-22

**Authors:** Maymouna Mourouvaye Payet, Nicolas A. Bonfils, Lisa Ouss, Lola J. Fourcade, Marie Touati-Pellegrin, Bernard Golse, Jérémie F. Cohen, Laure Woestelandt

**Affiliations:** ^1^Child and Adolescent Psychiatry Department, Necker-Enfants Malades Hospital, APHP, Université de Paris, Paris, France; ^2^Université de Paris, Faculty of Medicine, Paris, France; ^3^Department of General Pediatrics and Pediatric Infectious Diseases, Necker-Enfants Malades Hospital, APHP, Université de Paris, Paris, France

**Keywords:** attempted suicide, child, adolescent, inpatient care, family interventions, ethics

## Abstract

**Objectives:**

To assess practices of French psychiatrists regarding their management of children and adolescents with suicidal behaviors, focusing on the use of a separation protocol in which the youths are separated from their relatives.

**Methods:**

In 2017, we conducted an online cross-sectional survey of French psychiatrists caring for children and adolescents. Participants were asked to describe their practice of a separation protocol in children and adolescents admitted for suicidal behavior. Our main analysis followed a descriptive approach. We also explored whether participant characteristics were associated with the use of a separation protocol.

**Results:**

The response rate was 218/2403 (9,1%); 57.9 % of respondents worked in a University hospital, and 60% of respondents reported routinely hospitalizing children. A separation protocol was set up by 91.1% of survey participants (systematically 39.6%, on a case-by-case basis 51.5%). The mean age from which a separation protocol was indicated was above 11 years; 64% of participants reported a separation period of ≤ 48 h. The most common (87%) criterion cited for establishing a separation period was family relationship difficulties. The most common (80.9%) reason to justify the use of a separation protocol was to allow a better clinical assessment. Exploratory analyses did not identify any participant characteristics associated with the use of a separation protocol (*p* > 0.2 for all).

**Conclusion:**

The use of a separation protocol in children and adolescents admitted for suicidal behavior is a widespread practice in France, despite the deprivation of liberty it implies. This raises the question of the relevance and usefulness of such a practice.

## Introduction

In 2016, Europe registered the highest rate of death by suicide in the world: 15.4 for 100,000 population (1, 2). Suicide is the second leading cause of death among adolescents (3). Suicide attempts in children and adolescents are a major public health concern, recently reactivated by the COVID-19 pandemic. In France, 8.7 % of women and 5.5 % of men have reported a suicide attempt before their 15 years of age (4). Suicide rates in children and adolescents have dramatically decreased during the first COVID-19 lockdown (5), but have then increased (+299% in 2020) (6), for reasons that have not yet completely been understood. Pediatric Emergency Units and Pediatric Hospitalization Units have been directly exposed and overwhelmed by this increasing number of suicides. Practical questions concerning how to manage these young suicide attempters have emerged and pediatric teams have been forced to reexamine their ways to cope with this crucial and actual challenge.

In France, the latest clinical practice guidelines for managing child and adolescent suicide attempters were published in 1998 (7). The guidelines recommend that child and adolescent suicide attempters should systematically be referred to the emergency department (ED) for a triple somatic, psychological, and social evaluation. The psychological assessment must be performed by a psychiatrist within 24 h of admission and should explore the patient's pre-existing mental health status, biography, lifestyle, social and academic integration, and also the recent elements surrounding the suicide attempt. Risk and recidivism factors must be investigated, as well as the existence of any underlying psychiatric disorder. The guidelines highlight the importance of meeting the family in order to gather their vision of the suicidal crisis. The guidelines recommend that the families' agreement and participation in care are desirable and that efforts should be made to preserve the adolescent's independence. Finally, the guidelines mention that family therapy can be considered, but there is no clear guidance of a predefined protocol of care focused on adolescent-family interactions.

Despite the deprivation of liberty it implies, a French practice, or, at least, a Romance language country practice, is often proposed with suicidal children and adolescents: a *period of separation*. It refers to a protocol starting at the admission of children and adolescents presenting with suicidal behaviors (including suicide attempts and suicidal ideation), in which the child is kept away from his/her family and social environment for a given period, which can vary from one team to another (8). This intervention differs from seclusion or restraint. In Switzerland, a health care team has mentioned applying a separation protocol during 48 h in suicidal children and adolescents (9). Several French teams also apply a period of separation in the case of anorexia nervosa (10–13) and in adolescent psychiatry units (14). To our knowledge, this practice is not in use in other countries and is rarely questioned (15–17). We can hypothesize that the practice of separation results from the strong influence of psychoanalytical theories in French child and adolescent psychiatry (18). Adolescence has been described as the second step of the separation/individuation process (19) which implies a movement away from the family environment. In the USA, the outpatient model is preferred, underpinned by a different system of care. Of note, France has one of the most generous healthcare systems in terms of social security coverage (20).

To our knowledge, separation protocols are not in use in Anglo-Saxon settings and are not mentioned in any clinical practice guidelines. Also, we are not aware of any evaluation of the frequency or efficacy of this practice. The only reference we found concerned more seclusion/restraint in adults, and noted that “seclusion and/or restraint may be permitted … and are now considered safety measures of last resort”(21). In the USA, there are no existing guidelines or resources available for helping clinicians dealing with pediatrics mental health emergencies (22).

This survey aimed at exploring the practices of French psychiatrists regarding the management of children and adolescents presenting with suicidal behaviors, focusing on the use of a separation protocol.

## Materials and Methods

### Survey Participants

We conducted a web-based survey of French psychiatrists involved in the management of children and adolescents with suicidal behaviors. The inclusion criteria were as follows: (i) to be a hospital-based (including University, general, or specialized hospital) psychiatrist or a child and adolescent psychiatrist in France; (ii) to be, at the time of the study, an academic or non-academic practitioner or a resident. Pediatricians, ED physicians, and physicians of other specialties, medical students, nurses, and psychiatrists working in non-hospital facilities (e.g., day hospital, private practice) were not eligible.

### Survey Tool

We developed a self-administered questionnaire on the web-based platform SurveyMonkey®. The survey included thirty questions. The three first questions aimed to verify that the inclusion criteria were satisfied. The following eight questions collected socio-demographic data, organizational characteristics of the respondent's hospital department, and general information of their practices with suicidal youths. Then, 17 closed questions aimed to explore the separation protocol itself: practical implementation, eligibility criteria including age limits, duration, modalities, justification, format, and content of information delivered to the patient and his parents. Also, respondents explored their own experience of the separation protocol of children, parents, and caregivers. The two last questions collected general knowledge about suicidology. The questionnaire was piloted on two child and adolescent psychiatrists and one pediatrician and was revised accordingly. We used conditional branching in order to assign each respondent individually based on the previous answer. The English version of the tool is available (Supplementary File).

### Survey Invitations

Participants were invited through mailing lists. First, based on the official website of French academic hospitals, we identified hospitals that embedded child and adolescent health services and collected contact emails. Second, we used the national mailing list of the French Federative Association of Residents in Psychiatry (Association Française Fédérative des Étudiants en Psychiatrie, AFFEP). Third, we used the mailing list of a Child and Adolescent Psychiatry Association (Association des Psychiatres de secteur infanto-juvénile, API). Survey participants were invited by email. The survey was conducted over a 3-month period between June and September 2017.

### Statistical Analysis

We first performed a descriptive analysis of study participants and survey responses. To explore variability in responses, we undertook stratified univariable analyses by the following respondent characteristics: current status (residents vs. practitioners), current place of practice (academic vs. non-academic hospital), current medical field (general psychiatrists vs. child and adolescent psychiatrists), and the existence of consultation-liaison psychiatry on site (yes vs. no). Chi-square and Fisher's exact tests were used to compare qualitative variables. There was no specific sample size calculation for this study. The threshold for statistical significance was set at *p* < 0.05. Statistical analyses were performed using RStudio® version 1.0.153 (R Foundation for Statistical Computing, Vienna, Austria).

### Ethics

Participation in the survey was voluntary. A short paragraph was included at the beginning of the questionnaire to inform participants of the study's objectives and of the confidentiality of their responses. Consent was considered obtained by virtue of questionnaire completion. Data were collected anonymously, and participants had the right to access their answers. In accordance with French legal regulations, ethical approval was not required for this study.

## Results

### Participant Characteristics

**Figure 1:**
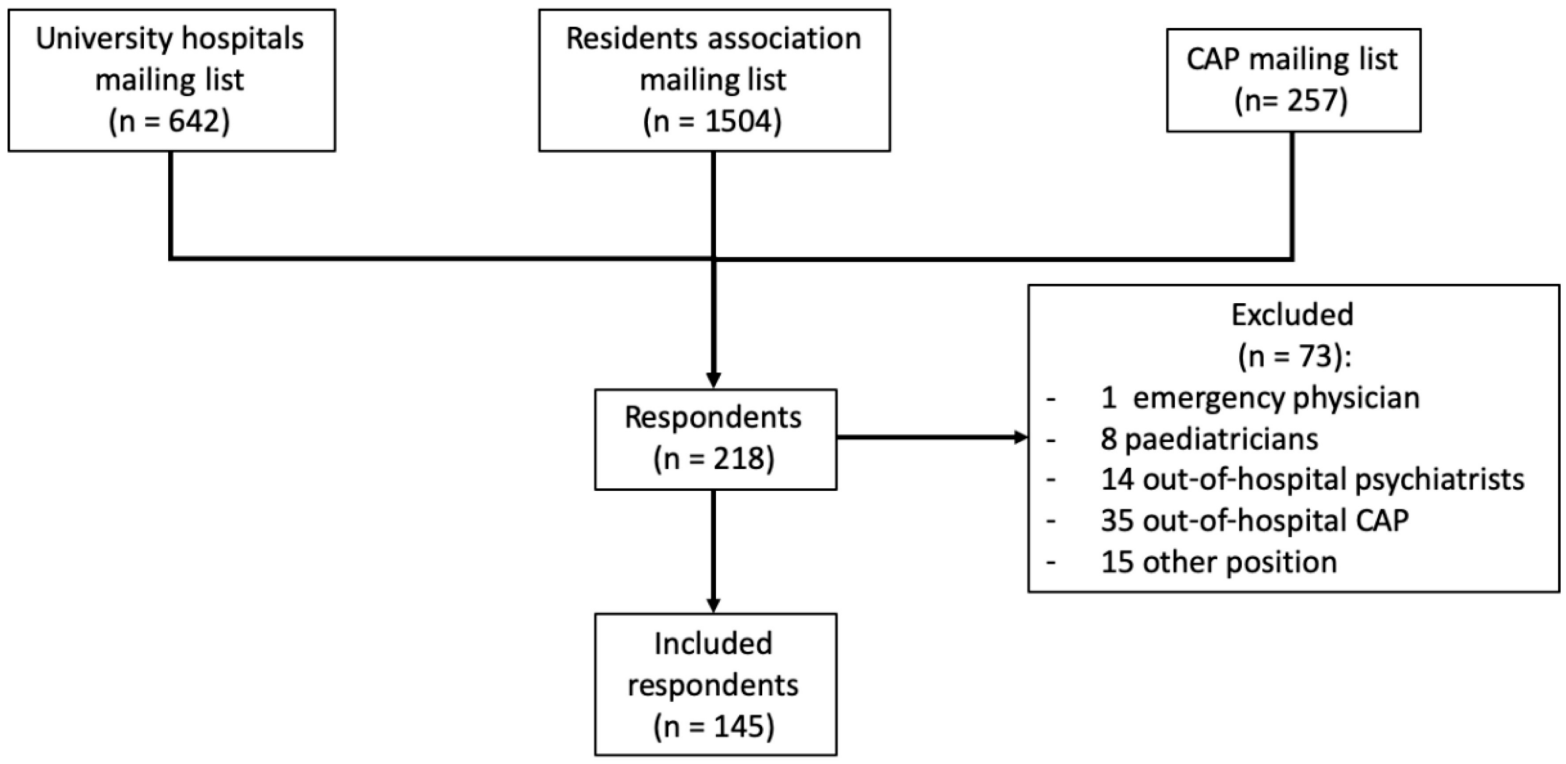
Flow chart of study participants.

Among 2,403 invitees, 218 (9.1%) respondents completed the questionnaire; 73 were excluded; 145 remained in the analysis (Figure 1). The average completion rate of the questionnaire was 79%. Respondents' socio-demographic data and characteristics are shown in Table 1.

**Table 1:** Summary of survey responses (*N* = 145).

**Characteristics of the psychiatrists who participated in the survey (*N =* 145)**	***n* (%)**
**Age, years**	
<30	39 (27.5)
30-39	52 (36.6)
40-55	31 (21.8)
>55	20 (14.1)
**Gender**	
Male	49 (34.3)
Female	94 (65.7)
**Current medical field**	
General psychiatry	37 (25.5)
Child and adolescent psychiatry	108 (74.5)
**Current professionnal status**	
Academic practitioner	20 (13.8)
Non-academic practitioner	83 (57.2)
Resident	42 (29)
**Practice setting**	
Non-academic hospital	61 (42.1)
University hospital	84 (57.9)
**Liaison child and adolescent psychiatry in place of practice**	
Yes	119 (83.2)
No	24 (16.8)
**Practice concerning child and adolescents suicidality**	
**Systematic admission for suicide attempt**	
Yes	83 (60.4)
No	55 (39.6)
**Systematic admission for suicidal ideas**	
Yes	27 (19.7)
No	109 (80.3)
**Hospitalization unit**	
General pediatrics	105 (78.5)
Specialized pediatrics	3 (2.2)
Child and adolescent psychiatry	87 (64.4)
Adult psychiatry	29 (21.5)
Post-Emergency unit	26 (19.3)
**Hospitalization duration for suicide attempt**	
3–5 days	68 (50)
>5 days	53 (39)
48 h	9 (6.6)
<48 h	6 (4.4)
**Hospitalization duration for suicidal ideas**	
3–5 days	60 (44.1)
>5 days	31 (22.8)
48 h	26 (19.1)
<48 h	19 (14)
**Separation protocol**	
Yes, always	53 (39.6)
Yes, on a case-by-case	69 (51.5)
No, never	12 (8.9)

### Hospital Admission Practices

Overall, 60.4% and 19.7% of respondents indicated a systematic admission for suicide attempters and for children and adolescents with suicidal ideation, respectively. Most respondents indicated that they usually referred young patients with suicide ideation or suicide attempt to a general pediatric unit or to a child and adolescent psychiatry unit (77.5 and 64.4%, respectively). Regarding the duration of hospital admission for adolescents with a suicide attempt, 50% of respondents reported 3–5 days of hospitalization, 39% a duration of more than 5 days, 6.6% reported a 48-h hospitalization, and 4.4 % <48 h. For children and adolescents with suicide ideation, 44.1% reported 3–5 days of hospitalization, 22.8% a duration of more than 5 days, 19.1% reported a 48-h hospitalization, and 14% <48 h.

### Knowledge, Attitudes, and Practices Regarding the Use of a Separation Protocol

**Figure 2:**
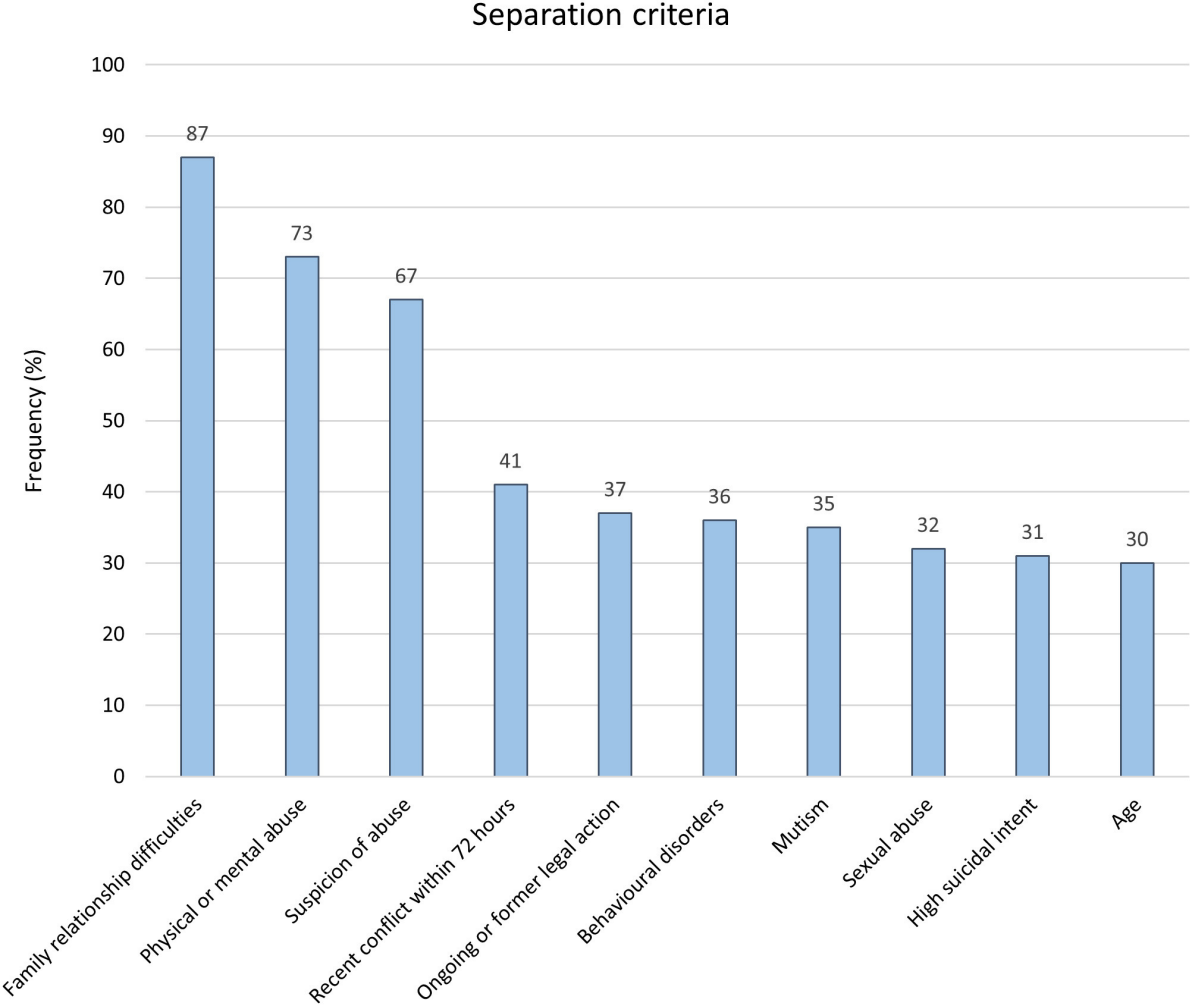
Ten most commonly cited separation criteria used to indicate a separation protocol.

A separation protocol was set up systematically by 39.6% of respondents, and on a case-by-case basis in 51.5%, for a total of 91.1%. The five most common separation criteria, when set up on a case-by-case basis, were family relationship difficulties, physical or mental abuse, suspicion of abuse, recent conflict within 72 h, ongoing or former child protection action (Figure 2). A separation protocol was indicated for patients with a mean (+/– standard deviation) age of 11.3 (+/–1.98) years. More than half of separation protocol practitioners (51.4%) reported a period of 24 to 48 hours of separation. Practical arrangements consisted of no exit allowed from the hospitalization unit (80.2%), no visit allowed (72.3), and no phone call (64.4%). A secured room (without items allowing suicide, such as sharp objects; regular supervision) was prescribed by 26.3% of the respondents, while 27.7% prescribed hospital pajamas. For 20.8% of respondents, only parental visits were allowed during the separation period.

Reasons given to justify the use of a separation protocol were to allow for a better clinical assessment (80.9%), to separate the child from a potentially harmful environment (62.9%), and to allow for time for reflection (48.9%). In total, 59.6% of survey respondents declared being aware of similar separation protocols in France, and 4.8% abroad. Interestingly, 15.4% of respondents reported knowledge of French recommendations on this practice (while there are none), and 4.8% reported being aware of international recommendations for separation protocols (while we are not aware of any). A written and oral information regarding the separation protocol was given to the parents in 37 % of cases. Most of respondents indicated an exclusive oral information (67%). The information given to children was mostly delivered orally too (81%).

### Exploratory Analyses of Factors Associated With Separation Practice

There was no significant difference in separation practice by participant characteristics (Table 2), including practitioner age, current professional status, practice setting, current medical field, and presence of a liaison team (*p* > 0.2 for all univariable analyses).

**Table 2:** Association between participant characteristics and the practice of separation (*N* = 134).

**Participant characteristic**	**N (%)**	**Separation**		**Odds ratio (95% CI)**	P^***^
		Yes^*^, Number (%)	No^**^, Number (%)		
**Age, years**					
<39	85 (63)	75 (88)	10 (12)	0.32 (0.03–1.61)	0.21
≥40	49 (37)	47 (96)	2 (4)	*Reference*	
**Current status**					
Senior practitioner	98 (73)	90 (92)	8 (8)	1.41 (0.29–5.67)	0.73
Resident	36 (27)	32 (89)	4 (11)	*Reference*	
**Practice setting**					
Non-academic hospital	54 (40)	50 (93)	4 (7)	1.39 (0.35–6.64)	0.76
Academic hospital	80 (60)	72 (90)	8 (10	*Reference*	
**Current medical field**					
Psychiatrist	33 (25)	31 (94)	2 (6)	1.70 (0.33–16.77)	0.73
Child and adolescent psychiatrist	101 (75)	91 (90)	10 (10)	*Reference*	
**Liaison child and adolescent psychiatry in place of practice**					
Yes	115 (86)	105 (91)	10 (9)	1.24 (0.12–6.57)	0.68
No	19 (14)	17 (89)	2 (11)	*Reference*	

* *Always or on a case-by-case basis*.

** *Never*.

*** *2-sided Fisher's exact test*.

## Discussion

In this national survey of French psychiatrists, we firstly found that separation protocols, in which child and adolescent suicide attempters are separated from their relatives, is a very common practice in France, even for relatively young adolescents. Then, we found no association between participant characteristics and separation practice, suggesting that such a practice is relatively homogeneous in the French setting and considered as “obvious”, and seems to be rarely questioned. The most common separation criteria were family relationship difficulties (87%), physical or mental abuse (73%), suspicion of abuse (67%), recent conflict within 72 h (41%) and ongoing or former child protection action (37%; Figure 2).

This survey raises two important questions. How to manage hospitalization after children and adolescent suicidal behavior? As expected, systematic admission was higher for suicide attempts than for suicide ideation. Reported rate of hospitalization was 60.4% of suicide attempts, which is relatively low, considering that French guidelines recommend systematically admitting all child and adolescent suicide attempters for a somatic, psychological, and social assessment (7). This low admission rate could be explained by the lack of beds in pediatric hospitals, notably in child and adolescent psychiatry units. The results may also reflect the practice of psychiatrists, but not that of ED physicians and pediatricians. Adolescents with suicidal ideas are hospitalized, but after a psychiatric consultation in the ED, when possible, and only the more severely depressed adolescents, or with strong suicidal ideas. Future studies should aim at better documenting the outcomes of the ambulatory management of children and adolescents seen in the ED for suicidal reasons (suicide attempt or suicide ideation). Directions have been proposed to prevent suicide, distinguishing factors that predict ideation from those that predict suicide attempts, in the framework of ideation-to-action (23).

Respondents declared that most patients with suicide attempt or ideation were admitted to departments of general pediatrics (78.5%). However, hospitalization rates in child psychiatry units were higher than expected, 64.4%, as we know that very few beds are available to welcome children after a suicidal behavior. An unexpected outcome is the relatively frequent use of adult psychiatric services (21.5%), which probably reflects the insufficient provision of child psychiatry care, or even its non-existence in some remote rural areas. The low use of specialized pediatric units could reflect that such units are a “second choice” in case there is no bed available in the department of general pediatrics, and could also be explained by the low occurrence of specific organ damage resulting from the suicidal act. The duration of hospitalization, frequently between 3 and 5 days, is consistent with the practices observed in our hospital, but is shorter than the one-week admission recommended by French guidelines.

But the main question concerns the relevance and usefulness of a practice of separation of children and adolescents from their family after admission to the hospital for suicidal behavior. When applied on a case-by-case basis, a separation protocol was supported by three types of criteria: criteria related to the child-environment link (environmental aspects: family relational difficulties, proven or suspected physical or psychological abuse, recent conflict within 72 h before the suicidal act, and ongoing or former child protection actions), criteria related to the patient's current psychiatric condition (synchronic elements: behavioral disorders, mutism, and strong suicidal intentionality), and the least cited criteria related to the child, diachronic elements (mainly past history of psychiatric disease).

The absence of any significant difference in separation practice by participant characteristics (current status, age, practice setting or even the current medical field) shows that this practice is not based on objective evidence and seems to be taken for granted in France.

To our knowledge, the practice of separation periods for child and adolescent suicide attempters has not yet been the object of clinical research. One Swiss study mentioned a separation protocol in this indication (9). It focused on the traumatic dimension of the adolescent's suicidality. Hospitalization in an intensive care unit confirms the “rupture” with the surrounding world that the patient has put into action through his suicidal act. This time of rupture is a controlled one. This idea of “decompression chamber” is materialized by the requirement that the patient has no contact with the outside world (telephones, outings, or visits) during the first 48 h of hospital stay (9).

It can be considered that full-time hospitalization in child psychiatry helps the adolescent to rearrange the boundaries with its environment. The parents are kept away in the reality but their links with the adolescent are worked through during the separation (24). A recent qualitative study investigated the experience of adolescents hospitalized in child and adolescent psychiatry departments (25). Separation feared, or even refused by the adolescent and sometimes the parents, can appear beneficial in the institution, but also sometimes very long afterwards. Another study collected testimonies of teenagers themselves, and showed that this distancing was beneficial to them. They also found that adaptation was possible and that a visit was arranged before the end of the period provided for when a child was having difficulty coping with the separation (26).

The separation protocol can be considered as an environmental care program, which focuses on the ED-post ED transition. The suicidal act corresponds to a failure of mentalization. Like some psychotherapeutic programs, the separation protocol seems to aim at providing the child or adolescent with a space where he can put into words what he has previously put into action. Unlike Mentalization Based Treatment (MBT), which is psycho-dynamically inspired, separation practices can be part of brief interventions that focus on the post-ED transition (27). Under no circumstances would a separation protocol replace long-term psychotherapeutic work, with which it must be associated.

But some arguments are against separation protocols. The stakes of such a separation practice are, above all, ethical. In France, a 1983 law introduced the establishment of civil servants who would rule on places of deprivation of liberty, particularly in psychiatric hospitals, under the supervision of a Controller General of Places of Deprivation of Liberty (CGLPL). This Controller is responsible for ensuring the application of the fundamental rights of minors in places of deprivation of liberty, including hospitals and psychiatry units. Concerning pediatrics wards, the last report of the CGLPL advocates for a flexible visiting regime, with wide access to parents, siblings, and friends, without age limit, as long as they are present in reasonable numbers and at reasonable times (26). The only limit indicated is the need not to interfere with medical care or disturb other patients. This limit, initially conceived of for a somatic universe, takes a particular turn for patients hospitalized for psychiatric reasons, in pediatric units where pediatricians are not used with familial separation. The 2017 report shows that in total contradiction with this law, the internal rules of most of the units provide for the suspension of all external links, including relatives, for a period generally ranging from 2 days to 1 week.

At the European level, Estonia has published recommendations on the issue of minors hospitalized in adult wards. They encourage young people to maintain relations with the outside world. But the report of the European Network of Ombudspersons for Children do not mention parent-child separation (28).

Finally, the place of parents and family in the treatment of child and adolescent psychiatric symptoms and diseases is changing, being more open to parents close involvement and cooperation (29). Furthermore, the qualitative sudy mentioned above showed that sometimes, separation was considered as traumatic, and may aggravate the adolescent' despair and difficulties (25).

We are thus confronted with two incompatible logics: to respect minor rights, parents' demands, and systematically propose separation protocols, which are supported by previous arguments. The latest French report on the fundamental rights of minors in mental health institutions states that restrictions on visits must be ordered by the physician in charge; they must be tailored in accordance with therapeutic needs. The benefits and the risks deserve further investigation.

### Strengths and Limitations

This study is the first to explore the management of an existing separation practice in France and to explore factors potentially associated with its use. This opens up perspectives for the harmonization of care. The main limitations of the study lie in the recruitment of survey participants. The mailing lists were not exhaustive, and the participants were included on a voluntary basis. In addition, it is difficult to assess the representativeness of the sample given the modest response rate. No formal sample size calculation was performed, and we simply aimed at obtaining the maximum number of responses. Our exploratory analyses of factors associated with separation practice did not allow us to identify statistically significant differences but they may have been underpowered. The low numbers of respondents in certain subgroups did not allow us to draw firm conclusions regarding associations between participant characteristics and the practice of separation. Also, we followed a pre-respondent approach and cannot exclude that several participants came from the same site, for a potential center effect. But we can hypothesize that only psychiatrists involved in adolescents' hospitalizations for suicide or suicidal intentions did respond. The low rate of respondents (9,1%), including a majority child and adolescent psychiatrists, is higher than the rate of child and adolescent psychiatrists among psychiatrists (3,8%) in France. A possible limitation is the tendency of social expectancy among participants of questionnaire surveys. We tried to reduce this influence using an anonymous approach.

## Conclusions

Our study highlights a reality of practices of separation in children and adolescents admitted for suicidal behavior. To our knowledge, the effectiveness of this separation practice has never been studied. The question of the deprivation of liberty involved in the prescription of such a systematic separation protocol is critical, even if the separation period is short. Beyond the justification of our practices with patients and their families, and their medico-legal implications, a deeper reflection must be undertaken. Studies should be conducted to evaluate the effectiveness of such separation protocols, particularly on recurrences of suicidal attempts. The development of a decision-making tree, using the Delphi method, for example, by a committee of experts would make it possible to harmonize practices in France, and question practices in other countries, while considering the uniqueness of each child, each family and each socio-cultural context.

## Data Availability Statement

The original contributions presented in the study are included in the article/Supplementary Material, further inquiries can be directed to the corresponding author.

## Author Contributions

MM, LW, and JC contributed to conception and design of the study. LF, MT, and BG contributed to conception of the study. NB performed the statistical analysis. MM, LO, and JC wrote the first draft of the manuscript. All authors contributed to manuscript revision, read, and approved the submitted version.

## Conflict of Interest

The authors declare that the research was conducted in the absence of any commercial or financial relationships that could be construed as a potential conflict of interest.

## Publisher's Note

All claims expressed in this article are solely those of the authors and do not necessarily represent those of their affiliated organizations, or those of the publisher, the editors and the reviewers. Any product that may be evaluated in this article, or claim that may be made by its manufacturer, is not guaranteed or endorsed by the publisher.
