# Supplementary material for: Separation Practices in Children and Adolescents Admitted for Suicidal Behavior: A National Survey of French Psychiatrists
Source: Front Pediatr. 2022 Jul 22;10:860267. doi: 10.3389/fped.2022.860267 (PMC9352949; doi:10.3389/fped.2022.860267)
Supplement: Supplementary file 1 [file Data_Sheet_1.PDF]

Appendix 1. Survey Questionnaire

In current practice of child psychiatry, it is common to propose a period of separation or isolation (which is sometimes imperative), during the care of the suicidal child or adolescent. By "separation", we mean the distancing of the surrounding environment, namely the entourage, when it exists, family and / or friends.
Our study focuses more specifically on the evaluation of this practice for suicidal patients (who have attempted suicide or have presented suicidal ideation), aged less than 15 years and 3 months.
This questionnaire proposes to make an inventory of knowledge and practices concerning this separation in the pediatric units and child psychiatric hospital departments in France.
The collection of this information is anonymous. However, we need to verify that the inclusion criteria are met. The first questions are dedicated this part.

1. Are you currently working as...?
(Your position (senior or resident) will be asked in question 2)
o Psychiatrist
o Hospital psychiatrist
o Child psychiatrist
o Hospital child psychiatrist
o Pediatrician
o Hospital pediatrician
o Emergency doctor
o Doctor from another specialty
o Other (specify)

2. What is your current position?
o Medical clerk
o Medical resident
o Faculty Senior practitioner (Prof, Assistant Prof, Associate Prof…)
o Non-Faculty Senior practitioner (PH, contractual PH, Associate Practitioner)
o Other (please specify)

3. Where are you currently working?
o In a CHU (University Hospital Center)
o In a General Hospital Center
o In a Specialized Hospital Center, in a Psychiatric Clinic, or in a Child Psychiatric Clinic
o In an extra-hospital setting: private practice, CMP, CMPP, CATTP, HDJ

4. Does your hospital have a psychiatric liaison activity with children and adolescents?
o Yes
o No

5. Are you…?
o A man
o A woman

6. How old are you (in years)?
o Under 30
o Between 30 and 39
o Between 40 and 55
o Over 55

7. In your facility, are suicidal children and adolescents (ie, who have attempted suicide) systematically hospitalized?
o Yes
o No

8. In your facility, are children and adolescents with suicidal ideas (ie, who have expressed suicidal thoughts or plans, but no attempt) systematically hospitalized?
o Yes
o No

9. When a child or adolescent, with suicidal thoughts or plans or suicidal behavior, is hospitalized, in what type of unit is the patient admitted? (Multiple choices possible)
o General Pediatrics
o Specialized Pediatrics (Neuro-pediatrics, Pneumo-pediatrics, etc.)
o Short-term unit or any other post-emergency unit
o Child psychiatry
o Adult psychiatry

10. In your opinion, most often, how long are suicidal children or adolescents (ie, who have attempted suicide) hospitalized in your facility?
o < 48 h
o 48 h
o 3 to 5 days
o > 5 days

11. In your opinion, most often, how long are children or adolescents with suicidal ideas (ie, who have expressed suicidal thoughts or plans, but no attempt) hospitalized in your facility?
o < 48 h
o 48 h
o 3 to 5 days
o > 5 days

12. Is a period of separation from the environment proposed during the hospitalization of children and adolescents with suicidal ideas or attempts?
o Yes, always
o No, never
o Sometimes, on a case-by-case basis

13. What are the criteria that motivate separation in your practice? (Multiple choices possible)
o Age of patient
o Male gender
o Female gender
o Current or former educational measure (AED type)
o Current or former judicial measure (AEMO type, placement, OPP)
o Somatic consequences requiring medical care in pediatrics
o Somatic consequences requiring medical care in intensive care
o Family relationship difficulties
o One (or more) personal history of suicidal attempt
o One (or more) personal history of psychiatric disease
o One (or more) family history of suicidal attempt
o One (or more) family history of psychiatric disease

o Intentionality muteness of the child or adolescent
o Substance abuse (eg, alcohol, cannabis)
o Risky sexual behavior
o Sexual abuse
o Proven psychological or physical abuse
o Suspicion of psychological or physical abuse
o Argument of the child or adolescent in the 72 hours preceding the act (eg, with family, friends)
o Behavioral disorders (eg, runaways, thefts, hetero-aggressiveness)
o Other (please specify)

14. If age is one of the criteria, from what age do you start proposing separation?
o 5 years old
o 6 years old
o 7 years old
o 8 years old
o 9 years old
o 10 years old
o 11 years old
o 12 years old
o 13 years old
o 14 years old
o 15 years old
o The presence of physical signs of puberty determines the proposal of a separation protocol
o Neither age nor physical signs of puberty determine the proposal of a separation protocol

15. Most often, if the separation is started, how long does it last?
o < 24 h
o 24 h
o Between 24 and 48 h
o 48 h
o Between 48 and 72 h
o Between 72 hours and one week
o One week
o > One week

16. If separation is started, what are the practical arrangements? (Multiple choices possible)
o “White room” (inventory, no personal objects, no dangerous objects)
o Putting on hospital pyjamas
o No visits allowed
o Only parents’ visits allowed
o No telephone calls allowed
o No authorization to leave the hospital ward
o No participation in ward activities and distractions such as games with peers, in-hospital school, clowns.
o Other (please specify)

17. In your opinion, why is this separation established? (several choices possible)?
o Because it is recommended in clinical practice guidelines
o To allow a better assessment of the clinical situation
o To separate the child from a potentially harmful environment
o To allow the child to reflect on his gesture
o To “mark the occasion”

o I don't know
o Other (please specify)

18. Are you aware of… (Several possible)
o French clinical practice guidelines regarding separation
o International clinical practice guidelines regarding separation
o Specific goals of this separation practice
o Similar protocols in French hospitals other than yours
o Similar protocols, at the international level
o None of the above
o Other (please specify)

19. From your experience, what is the general experience of the patient during the implementation of this period of separation?
o Fairly good
o Fairly average
o Fairly bad
o Don't know

20. From your experience, what is the general experience of the patient at the end of this period of separation?
o Fairly good
o Fairly average
o Fairly bad
o Don't know

21. From your experience, what is the general experience of parents when this period of separation is announced?
o Fairly good
o Fairly average
o Fairly bad
o Don't know

22. From your experience, what is the general experience of the parents at the end of this period of separation?
o Fairly good
o Fairly average
o Fairly bad
o Don't know

23. From your experience, what is the general experience of caregivers at the beginning of the period of separation?
o Fairly good
o Fairly average
o Fairly bad
o Don't know

24. From your experience, what is the general experience of caregivers at the end of the separation period?
o Fairly good
o Fairly average
o Fairly bad

o Don't know

25. How is the information about this period of separation given to the parents?
o Oral information
o Written information
o Both

26. What is the content of this information given to parents?
o The practical modalities of the separation regarding visits, calls, activities
o The duration of the separation
o The theory which underlies the practice of the separation
o The goals of the separation
o None of these proposals
o Other (Please specify)

27. How is information about this period of separation given to the child or adolescent?
o Oral information
o Written information
o Both

28. What is the content of this information given to the child or adolescent?
o The practical modalities of the separation regarding visits, calls, activities
o The duration of the separation
o The theory which underlies the practice of the separation
o The goals of the separation
o None of these proposals
o Other (Please specify)

29. Do you assess suicidal intentionality using the Beck Scale in adolescents?
o Yes, systematically
o No, never
o Sometimes, it depends on the case
o I do not know this tool
o Other (please specify)

30. Have you ever read the ANAES (National Agency for Accreditation and Health Evaluation) 1998 guidelines regarding the hospital care of suicidal adolescents?
o Yes, systematically
o No, never
